# Anaplastic Pleomorphic Xanthoastrocytoma: A Rare Variant of Astrocytoma

**DOI:** 10.7759/cureus.23060

**Published:** 2022-03-11

**Authors:** Khalilalrahman Alshantti, Chandran Nadarajan, Mitchell Modi Mijol, Anani Aila Mat Zin

**Affiliations:** 1 Department of Radiology, School of Medical Sciences, Universiti Sains Malaysia, Health Campus, Kubang Kerian, MYS; 2 Department of Pathology, School of Medical Sciences, Universiti Sains Malaysia, Health Campus, Kubang Kerian, MYS

**Keywords:** neuroradiology, neurosurgery, astrocytoma, pleomorphic xanthoastrocytoma, anaplastic pleomorphic xanthoastrocytoma

## Abstract

Pleomorphic xanthoastrocytoma (PXA) is a rare glioma that affects 1% of astrocytic tumors. It most commonly affects children and teenagers. PXA cases with an anaplastic histopathological subtype have been reported in recent years. It was recently classified as a separate subtype in the 2016 World Health Organization (WHO) classification of central nervous system (CNS) tumors. A 48-year-old healthy gentleman presented with progressive right upper limb weakness. CT and MRI of the brain were done, which showed an intra-axial supratentorial tumor. A diagnosis of high-grade glioma was initially made based on its imaging features. The histopathological study came back as anaplastic pleomorphic xanthoastrocytoma. After a discussion with the neurosurgical and oncology teams, a decision was made to treat the patient with radiotherapy. In this case report, we describe a rare case of PXA with anaplastic characteristics.

## Introduction

The World Health Organization (WHO) added molecular characteristics to its classification of central nervous system (CNS) tumors in 2016, redefining and articulating how CNS tumors should be constructed in the molecular era. Pleomorphic xanthoastrocytoma (PXA) is a low-grade astrocytic neoplasm that accounts for 1% of astrocytic tumors [1], with a relatively good prognosis [2]. In 1979, Kepes et al. first identified PXA with anaplastic characteristics [3], and it was recognized as a separate brain tumor in 1993. Prior to 2016, it was classified as a WHO grade II tumor along with PXA. The 2016 WHO classification divides PXA and anaplastic pleomorphic xanthoastrocytoma (APXA) into two distinct entities, grade II and grade III variants WHO, respectively. Anaplastic pleomorphic xanthoastrocytoma (APXA) is classified as five mitoses per 10 high-power fields. For individuals with PXA, a gross total resection without adjuvant therapy may be sufficient. However, for those with APXA, complete resection with adjuvant therapy is necessary [4]. As a result, MRI helps differentiate the two types of tumor before surgery, benefiting patient management and prognosis prediction. Conventional MRI provides valuable information for determining the aggressiveness of a tumor and grading it. Here, we present a case of PXA with anaplastic features with its MRI features and management compared to PXA.

## Case presentation

**Figure 1:**
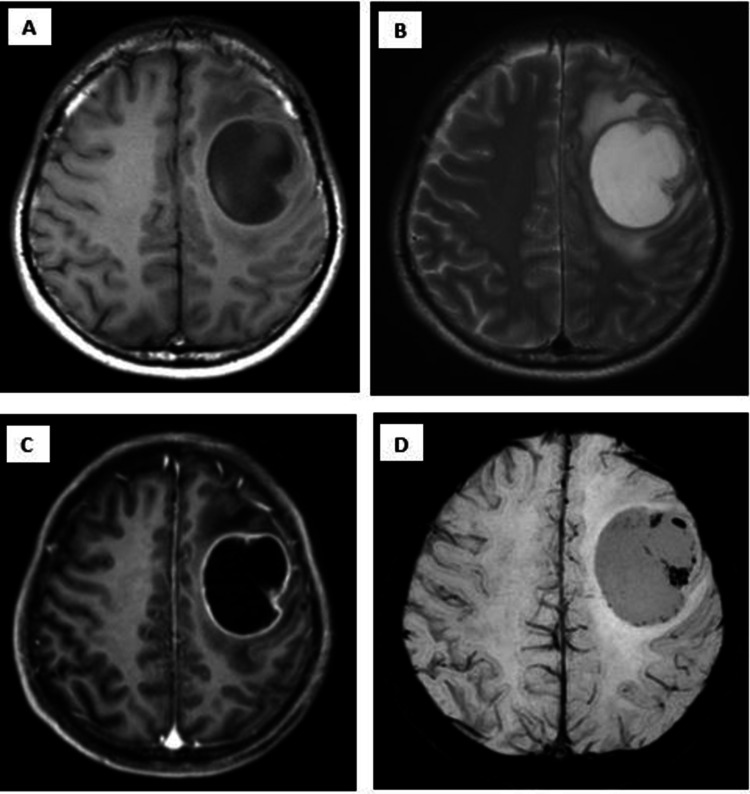
Left frontal lobe intra-axial mass. A-D) Left frontal lobe intra-axial mass which appears hypointense on T1 (A), hyperintense on T2 (B) and peripherally enhancing post gadolinium (C). Blooming artifact seen on susceptibility-weighted imaging (SWI) (D) suggestive of bleed.

**Figure 2:**
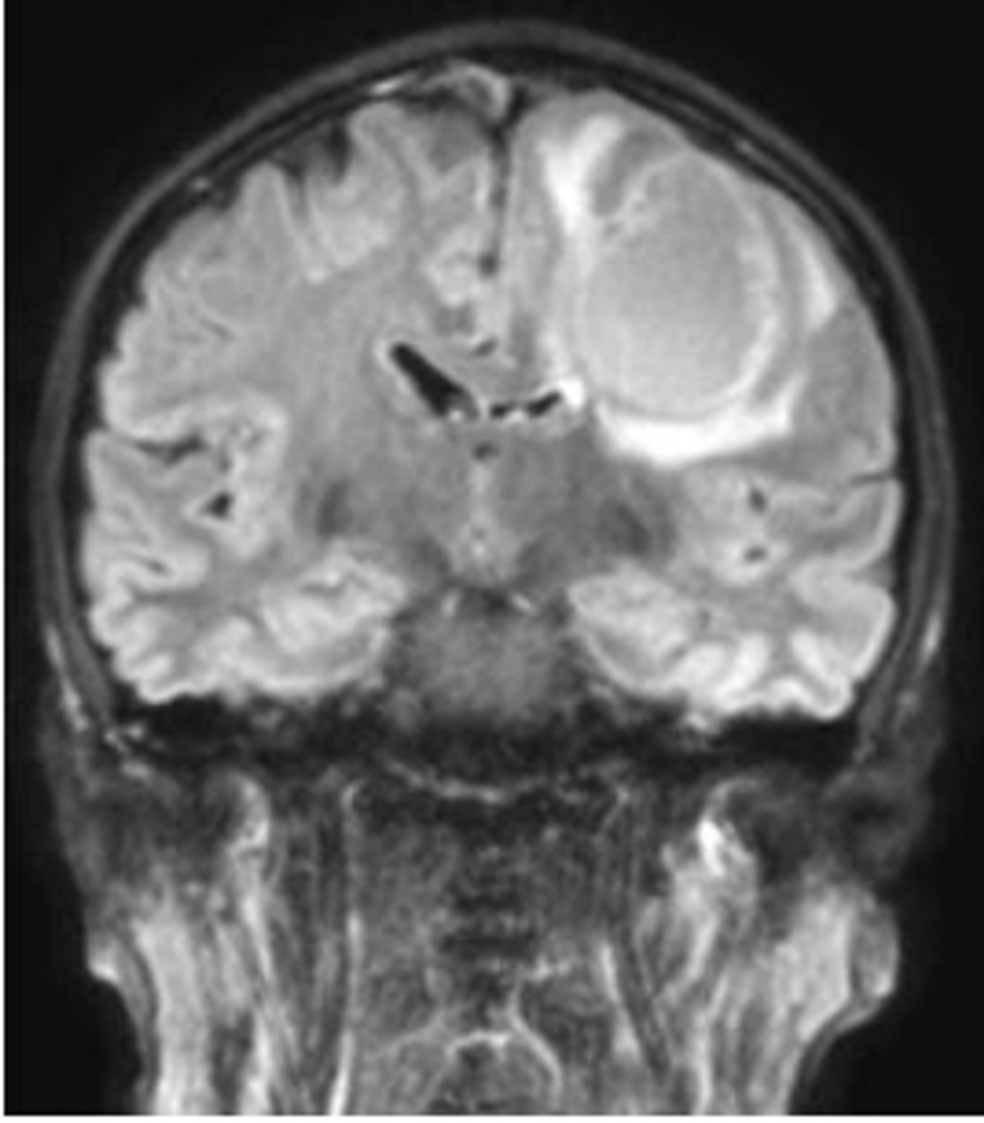
Lesion is partially suppressed on coronal fluid-attenuated inversion recovery (FLAIR).

**Figure 3:**
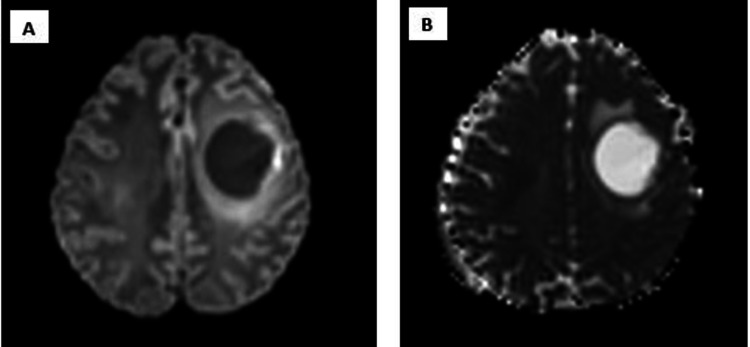
DWI/ADC sequences show restricted diffusion. DWI (A). ADC (B). DWI: diffusion-weighted imaging, ADC: apparent diffusion coefficient.

**Figure 4:**
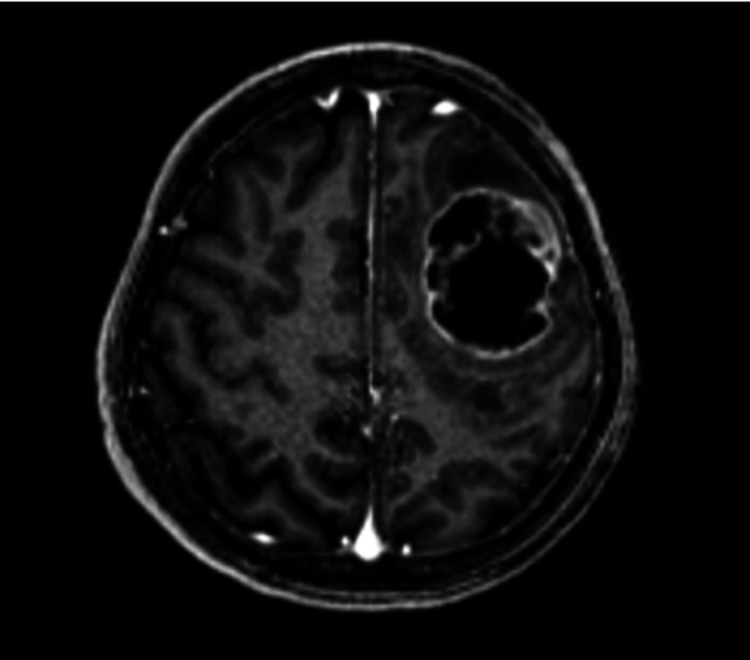
Enhancing solid component at the left lateral region of the mass with multiple thick irregularly enhancing septations at the superior region.

**Figure 5:**
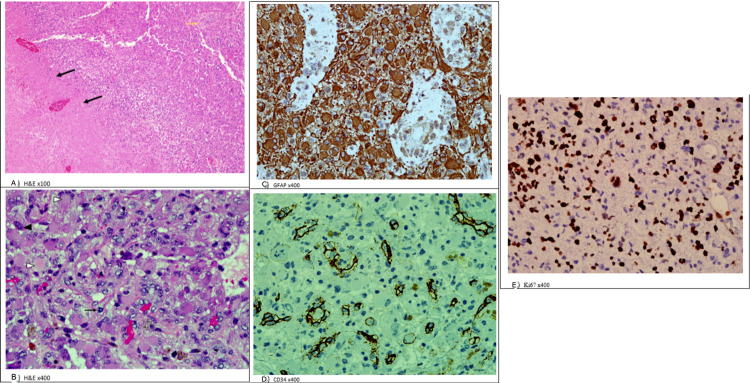
Stain of brain tissue. A, B) Hematoxylin and eosin stain (H&E) of brain tissue demonstrates diffuse infiltration of tumor cells with areas of pseudopalisading necrosis (A, black arrow) and perivascular endothelial proliferation (A, yellow arrow). The tumors have pleomorphic nuclei, some of which show bizarre giant tumor cells (B, black arrow head). Xanthomatous cells (B, white arrow head). Mitoses are noted (B, black arrow). C) Glial fibrillary acidic protein (GFAP) immunostain highlights the lipid vacuoles of xanthomatous cells. D) Cluster of differentiation (CD-34) immunostain shows focal positive expression with ramified process. E) The marker of proliferation (Ki67) immunostain shows high tumor proliferation index (50%).

A 48-year-old male with no prior history of medical illness presented with evolving right upper limb weakness for one month prior to admission. His family members noted that he was also less talkative during this period. No family history of malignancy or mental illness was reported. On first assessment, his Glasgow Coma Scale (GCS) was 15/15 with preserved high cortical functions. Muscle strength of the right upper limb was reduced to 4/5 with the presence of hyperreflexia. The power of other limbs was preserved with intact sensation. Cranial nerves function was normal. CT and MRI of the brain were performed urgently. It revealed a well-defined peripherally enhanced left frontal lobe intra-axial cystic mass associated with surrounding vasogenic edema. It measured approximately 4.4 cm x 3.8 cm x 4.1 cm (anterior-posterior (AP) x width (W) x craniocaudal (CC)) (Figures 1A-1D, 2) with restricted diffusion on diffusion-weighted imaging (DWI)/apparent diffusion coefficient (ADC) of the solid component (Figures 3A, 3B). The presence of solid components is noted at the superior left lateral region of the mass, which enhances avidly post gadolinium (Figure 4). Multiple thick irregular enhancing septations were seen at the superior aspect of the mass. There is a mass effect on the surrounding sulci and ipsilateral frontal horn of the left lateral ventricle. No other lesions were noted. There was no leptomeningeal enhancement. Based on the clinical and radiological features, high-grade glioma was diagnosed. Left frontal craniotomy and biopsy were performed. The histopathological examination revealed features of APXA (Figures 5A-5E). After a multidisciplinary consensus, gross total resection followed by radiotherapy was performed. Post-operation, the patient maintains the status quo with no worsening of symptoms.

## Discussion

Pleomorphic xanthoastrocytoma occurs in both men and women in their second decade of life. Most patients with APXAs were middle-aged in the research by She et al., with an average age of 47.7 years old at presentation time [5]. These tumors are frequently found in the cerebral hemispheres and do not involve the dura mater. Other desmoplastic neuroepithelial neoplasms include gliofibroma, gliosarcoma, desmoplastic infantile ganglioglioma and desmoplastic cerebral astrocytoma of infancy, which have been discovered to share some clinical, radiological and pathologic features with APXA [6]. Moreover, epithelioid glioblastoma can be difficult to distinguish from APXA because of similar histopathological features. MRI provides helpful information regarding tumor size, perilesional edema, infiltration, hemorrhage and necrosis. Previous case reports showed APXA has a significantly greater tumor size than PXA because of an increased mitotic rate by more than five mitoses per 10 high-power fields. APXA also has more significant tumoral enhancement and perilesional edema [5]. There have been multiple attempts to identify tools to differentiate between these two tumors, such as the ratio between DWI and ADC values, aiming to assess the differences between PXA and APXA based on nuclear to cytoplasmic ratio [7]. Another study reported that increased microvascular proliferation in anaplastic tumors increases the relative cerebral blood volume (rCBV) value, which is correlated with cellular proliferation in high-grade gliomas, and in the modern MRI techniques, rCBV is considered as a significant marker for tumor vascularity [8]. However, these case studies have a small sample size, making generalization difficult. Standard postoperative therapy has yet to be developed because of the rarity of PXA with anaplastic characteristics in the literature [9]. Variable treatment options and outcomes are revealed in a review by Patibandla et al., who gathered data from prior case reports [10,11]. Long-term control achieved with surgical resection and stereotactic radiation therapy has been reported [10]. The treatment of PXA with anaplastic characteristics has not been found to benefit from conventional radiotherapy or chemotherapy with novel medications, such as temozolomide. Stereotactic radiosurgery may be beneficial in the therapy of reversing progression, but more research is needed [11].

## Conclusions

APXA is rare and often has more aggressive radiological features which can mimic the high-grade astrocytomas than pleomorphic xanthoastrocytomas, but it is a distinct tumor type that needs to be identified and treated separately from PXA. Imaging and histopathological examination could guide the clinician to a correct diagnosis and treatment planning. 
